# Fluctuating capacity and advance decision-making in Bipolar Affective Disorder — Self-binding directives and self-determination

**DOI:** 10.1016/j.ijlp.2015.04.004

**Published:** 2015

**Authors:** Tania Gergel, Gareth S. Owen

**Affiliations:** a Department of Psychological Medicine, King's College London, Institute of Psychiatry, Psychology and Neuroscience, C/O Department of Classics, King's College London, Strand, London WC2R 2LS, UK; b King's College London, Institute of Psychiatry, Psychology and Neuroscience, Department of Psychological Medicine, UK

**Keywords:** SBD, self-binding (advance) directive, PAD, psychiatric advance directive, DMC-T, capacity to make decisions concerning treatment, Advance directives, Self-binding directives/Ulysses Contract, Precedent autonomy, Mania, Bipolar Affective Disorder, Decision making capacity

## Abstract

For people with Bipolar Affective Disorder, a self-binding (advance) directive (SBD), by which they commit themselves to treatment during future episodes of mania, even if unwilling, can seem the most rational way to deal with an imperfect predicament. Knowing that mania will almost certainly cause enormous damage to themselves, their preferred solution may well be to allow trusted others to enforce treatment and constraint, traumatic though this may be. No adequate provision exists for drafting a truly effective SBD and efforts to establish such provision are hampered by very valid, but also paralysing ethical, clinical and legal concerns. Effectively, the autonomy and rights of people with bipolar are being ‘protected’ through being denied an opportunity to protect themselves. From a standpoint firmly rooted in the clinical context and experience of mania, this article argues that an SBD, based on a patient-centred evaluation of capacity to make treatment decisions (DMC-T) and grounded within the clinician–patient relationship, could represent a legitimate and ethically coherent form of self-determination. After setting out background information on fluctuating capacity, mania and advance directives, this article proposes a framework for constructing such an SBD, and considers common objections, possible solutions and suggestions for future research.

## 1 Introduction: the dilemma — the case of P

P is a 48 year old man who used to work as an electrician and has a diagnosis of Bipolar Affective Disorder. Since early adulthood he has experienced manic episodes, lasting a few months each, as well as one severe depressive episode, during which he attempted suicide by trying to jump off a bridge. He has now had approximately twenty hospital admissions. When well he tries his best to get on with his life, maintaining close contact with two grown up children and pursuing an interest in music. Both P and those who know him regard him as entirely well and ‘himself’ when in remission, but as a “different person” when ill/manic, whose behaviour is utterly uncharacteristic and associated with major harms to his relationships, self-esteem, property and affairs. During one episode, for example, his flat was stripped of all property including his bed and his car was taken.

P is intelligent and, when well, has very good insight into his condition and its consequences. He is persuaded that medication has helped to treat manic episodes, but not that adherence to medication is helpful in preventing further episodes. Although his family recognise the early symptoms of an episode and often report these to his team, P is ordinarily resistant to treatment at these points and presents as in control of potential harms. Typically, he is considered below the threshold for compulsory treatment. This does occur, but is delayed until the situation has deteriorated significantly: “instead of them coming and getting me at the beginning, they'll leave me on the street, to get worse and worse, and worse and worse.” In P's retrospective view, compulsory treatment has started too late.

P is desperate to stop this happening. He can clearly identify key prodromal indicators and, for the last three years he has repeated a wish for early intervention with compulsory treatment under Section 3 of England's Mental Health Act (1983)^1^, as soon as the episode is beginning. P has repeated past experience of compulsory treatment. He says “I hate Section 3 anyway” and recognises that his proposed solution will be harrowing. Nevertheless, he maintains:

When becoming unwell treatment at home is not suitable for me. I need to be admitted to hospital under compulsory treatment, even if I seem to still have some control — otherwise I am likely to get overconfident and start getting involved with people and activities that disrupt my life.

For P this would be a way of using his retrospective knowledge of his illness, when he has capacity, to minimise its enormous personal cost and take back control over his life.

P is attempting to use his surrounding social, clinical and legal framework to enable a form of self-determination known as a self-binding (advance) directive (SBD) or Ulysses Contract, in order to take control over his illness and limit the damage it causes. He stipulates early warning signs that should both be understood to indicate the need for an assessment for compulsory detention and treatment, and should inform the judgement which is made. These include fast speech, irritability, and grandiosity, along with behaviours like “bible-bashing,” going travelling and talking about philosophical questions. His stipulations are based on extensive past experience.

At present, however, the UK and nearly all other jurisdictions have no established clinical or legal provision to support this form of self-determination, even though both P and his clinicians can see that pre-binding to accepting treatment before major deterioration ensued could avert enormous damage. Like others with his condition, he faces a future of knowing that further episodes will most likely come, but being powerless to protect himself from their devastating effects.

Psychiatric advance directives (PADS) are the focus of increasing debate, being mooted as a mechanism to enhance self-determination within psychiatry, and discussion has intensified in the wake of the UN's Convention on the Rights of Persons with Disabilities (2008) (CRPD). However, though welcomed at a theoretical level, this is not reflected in practice and, even where provision exists, their use is uncommon (Backlar, 1998; Gallagher, 1998; Jeste & Saks, 2006, p. 624; Sarin, 2012; Srebnik & Kim, 2006; Swanson et al., 2003; Varekamp, 2004). A frequently cited aim of PADs in general is to reduce the need for coercion through, for example, improving crisis management recommendations based on patients' past experiences and preferences (Henderson et al., 2004; Khazaal, Chatton, Pasandin, Zullino, & Preisig, 2009; Swanson et al., 2008; Thornicroft et al., 2013). By contrast, a primary outcome of SBDs is to enable the individual to request introduction of coercive interventions in the earlier stages of illness, in order to prevent themselves from engaging in damaging and risky behaviour as they deteriorate (Gremmen, Widdershoven, Beekman, Zuijderhoudt, & Sevenhuijsen, 2008). Within the context of broad concerns about psychiatric coercion and current interpretations of the CRPD, which reject entirely judgements of incapacity and use of coercion (2014), this central coercive aspect of SBDs might seem problematic. Nevertheless, we contend that they could represent an ethically coherent means of enhancing self-determination which is consistent with the broadly worded aims of enablement and empowerment espoused within the CRPD itself.

We propose a form of SBD based on decision-making capacity for treatment (DMC-T) and argue that SBDs could have the potential to allow bipolar patients increased control and damage limitation. We will examine: the ‘fluctuating capacity’ characteristic of severe Bipolar Affective Disorder; the limitations of existing legal types of advance directives in this context; how to address associated rights-based and clinical difficulties. Unlike many discussions surrounding advance decision-making, which stem from an ethical and legal perspective, our starting-point is firmly rooted in the clinical context.

There is no perfect solution for the management of manic episodes, which, all too often, bring enormous personal damage and eventual deprivation of liberty — in the words of Gremmen et al. “coercion and care do not easily go together (2008).” SBDs will always involve a process of cost/benefit analysis, with major concerns on both sides. The notion of voluntarily committing one's conscious and often very lucid self to being treated involuntarily can seem shocking, especially to those unacquainted with the realities of living with mania. Even to clinicians, whose experience of patients is often dominated by times of crisis, it can seem inconceivable that a patient when well will have both the desire and ability to engage in a self-binding process. However, when well, those attempting to navigate the minefield of life with bipolar disorder are left to struggle with both the damage from previous episodes and their fears for what future episodes might bring. An SBD, for those who wish to draft one, may well be the best available option, despite its costs (Gallagher, 1998). For the sake of individuals such as P, we argue that the legal and clinical community has a strong moral obligation to consider the feasibility of allowing the type of provision P requests (Gevers, 2002; Rosenson & Kasten, 1991; Sheetz, 2006; Winston, Winston, Appelbaum, & Rhoden, 1982).

## 2 Background

### 2.1 What do we mean by “fluctuating capacity” in bipolar disorder?

**Fig. 1:**
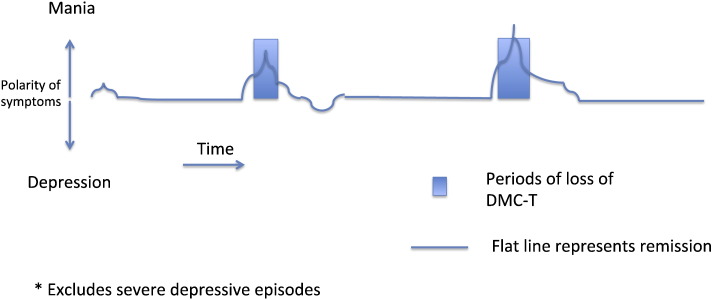
Fluctuating capacity and remission in mania*. *Excludes severe depressive episodes.

The term ‘fluctuating capacity’ is not recognised in statute, but has been used in secondary legislation, such as the Mental Capacity Act Code of Practice (Department of Constitutional Affairs, 2007) and case law^2^. It is associated with changes in DMC-T characteristic of the onset and recovery from episodes of a psychiatric disorder such as mania within bipolar disorder. A typical time course showing change in symptoms and change in DMC-T is shown in Fig. 1.

Generally, DMC-T is lost as an episode worsens and then returns with recovery. Episodes occur on a cyclical basis, with substantial periods of remission and full capacity in between. Given that onset and recovery are usually gradual, it can be difficult to determine the exact moment at which DMC-T is judged lost or regained.

Loss of DMC-T is extremely common in mania, probably more so than for any other psychiatric condition, and is often regained in about a month (Owen et al., 2011). Several studies have concluded that the majority of manic inpatients are lacking in DMC-T (Beckett & Chaplin, 2006; Cairns et al., 2005a; Owen et al., 2008). The most recent of these, which involved clinical interviews and structured DMC-T assessment, reported that 97% of those admitted with mania to a psychiatric ward, whether formally or informally, had impaired DMC-T (Owen et al., 2008). Importantly, loss of DMC-T in mania associates strongly with two characteristics. These are loss of insight – a clinical construct depicting self-awareness of mental state change and illness (Owen et al., 2009); and loss of appreciation – a legal construct depicting ability to apply information abstractly understood to oneself. By contrast, within general medical hospital patients, it is most usually cognitive abilities which are affected (Owen et al., 2013). Thus, despite concerns which are frequently raised (Breden & Vollmann, 2004; Gallagher, 1998; Tan & Hope, 2008; Weller, 2013, pp. 8–9), it should not be assumed: that determining the lack of DMC-T in psychiatry is generally problematic; that evidence of good cognitive skills within mania is indicative of DMC-T; or, that capacity assessment has an exclusively cognitive bias, rendering it unable to accommodate the type of impairments associated with mania or other affective disorders. Capacity assessment itself should take into account not simply narrow concepts of reasoning, but all the decision-making abilities recognised by law (Dunn et al., 2006; Kim, 2009; Lepping, 2011).

Fluctuating capacity is sometimes conflated with fluctuations in mental state and preference, which can occur during states such as mania. Mania is not a static mental state and an individual's presentation, preferences and opinions may change within it. Sometimes, these changes may appear to involve coherent patterns of reasoning and increased insight. Nevertheless, even if this makes the interpretation challenging, these “fluctuations” of mental state do not indicate the types of deep changes which occur as the manic episode begins or comes to its end.

Extremely important here is also the differentiation between DMC-T and the broader notion of legal capacity. There are multiple definitions of legal capacity. However, we are using the term “legal capacity” to denote an individual's capacity to hold rights on an equal basis with others and to be, as it were, visible to human rights law. We will argue below that this essential right does not fluctuate and must be both distinct from and unaffected by formal judgement of the lack of DMC-T.

### 2.2 Mania — key characteristics

Bipolar disorder, formerly known as manic depression, is a cross-cultural disorder, with high heritability (Bienvenu, Davydow, & Kendler, 2011; McGuffin et al., 2003). It is associated with high IQ early in life (MacCabe et al., 2010) and with addiction and suicide throughout life (Merikangas et al., 2008; Osby, Brandt, Correia, Ekbom, & Sparén, 2001). Although the condition typically includes both manic and depressive stages, we are focusing on mania, the stage most closely associated with fluctuating capacity. Manic episodes follow a cyclical pattern and the shift into mania is usually a gradual process of escalation. Very often, an individual experiencing mania will lack insight that they are unwell and be unaware of the risks of their behaviour. This, for example, is how P puts it:

you're just unwell at the time. I mean, I know the difference between being well and being unwell, but when you're under [mania] you don't know the difference…because everything seems so real you think that you are well and that everybody else is making a wrong diagnosis.

Mania itself can include a full range of symptoms found in other mental disorders and is usually characterised by a combination of relentless energy, elation and lack of both inhibition and doubt. P says:

before you go into hospital you're high and you're putting yourself at risk. You're putting yourself at risk because you're letting Tom, Dick and Harry into your house, who are robbing all your possessions, and I'm going onto the frontline and stuff like that, and challenging crack dealers, and stuff like that. And I put myself at risk by doing that. And even though you don't feel it at the time, that's what you're actually doing.

Decreased need for sleep is accompanied by a constant need for stimulation, which can manifest itself in extremes of spending, socialising, sexual activity and substance abuse.

While the subjective experience of mania is frequently one of extraordinary abilities and enlightenment, it is often accompanied by paranoia or other delusions, anger, agitation and claustrophobia. While P may believe himself to be an inspired scientist, who has read every book written in the past, present or future, he can also believe that hospital are monitoring him through a device in his head. For some, it can manifest itself as, or become, ‘mixed’ or ‘dysphoric’ mania, often regarded as the most dangerous of all disordered mental states in terms of suicide risk, with the energy, disinhibition and impulsivity of mania combined with the most negative and self-destructive aspects of depression. In her autobiographical account of living with bipolar disorder, the US psychiatrist Kay Jamison describes the moments leading up to a suicide attempt:

I can't think. I can't calm this murderous cauldron, my grand ideas of an hour ago seem absurd and pathetic, my life is in ruins and – worse still – ruinous; my body is uninhabitable. It is raging and weeping and full of destruction and wild energy gone amok. In the mirror I see a creature I don't know but must live and share my mind with. I understand why Jekyll killed himself before Hyde had taken over completely. I took a massive overdose of lithium with no regrets (1995, p. 115).

Jamison was discovered and survived. She has now written an SBD type agreement, requesting hospitalisation and treatment with ECT, even if unwilling, should such a situation recur (1995, p. 113).

Many people with bipolar can manage to find an effective and acceptable maintenance strategy over time, which can keep the condition under control using medication and other forms of lifestyle management. However, there are also many, like P, for whom a successful and realistic long-term strategy cannot be found (Berghmans & van der Zanden, 2012). Treatments which work at certain times of life or certain stages of the illness may be less successful long-term, or unfeasible due to side-effects. Episodes can be sparked by physical or mental stresses which are common in the course of ordinary life, such as sleep loss or disturbance, illness, pregnancy, overwork, excessive stress or alcohol and substance use. Without a perfect means of control, any of these factors can act as triggers for mania, leaving those with bipolar to live with a constant risk of future manic episodes and their collateral damage.

### 2.3 Advanced decision making — current law and practice

Advance decision-making can be thought of a codification of what Ronald Dworkin (1990, p. 226). Precedent autonomy is the idea that an individual's preferences when autonomous trump their preferences when lacking autonomy and that this can extend self-determination to incapacity^3^.

At present, there are three main models of advance decision making, recognised to varying degrees within legislation. The UK and many other jurisdictions recognise some type of advanced refusal of treatment, such as the Advanced Decision to Refuse Treatment (ADRT) within England's Mental Capacity Act (2005) (MCA). These can only be made when DMC-T is present and are designed to apply when DMC-T is absent, although they are subject to court approval and can be overturned by mental health legislation. They are, however, limited to treatment refusals and therefore have limited relevance for SBDs in a case such as P's.

An individual can also make an advance statement of wishes concerning treatment and management of future psychiatric episodes. Often termed ‘living wills’ or ‘instruction directives’ (Gallagher, 1998; Gevers, 2002), this model of advance decision-making comes closer to P's suggestions. Nevertheless, it is essentially non-binding and the legal informality would not authorise the compulsory treatment P has in mind. Instruction directives are widely supported, but there are implementation problems and the empirical evidence for effectiveness is not currently compelling (Campbell & Kisely, 2009; Fagerlin & Schneider, 2004; Sheetz, 2006; Thornicroft et al., 2013).

Finally, there are provisions, in many jurisdictions, for an individual with DMC to hand over decision-making to a proxy (e.g. a family member) at a time DMC is lost. In the MCA this is the Lasting Power of Attorney (LPA). While these are more often used for decisions concerning property and affairs, LPAs may also be constructed for healthcare decisions, although they are essentially untested in relation to psychiatric disorders such as bipolar. When DMC-T is lost a healthcare professional must, in effect, seek treatment permissions from the LPA in place of their patient. Where treatment involves coercion or deprivation of liberty mental health laws prevail which make no formal use of these proxy decision-making structures.

Existing legislation surrounding advance decision making is not a good fit for bipolar. In general, the basic forms have been constructed to manage end of life situations and disorders such as Alzheimer's Disease or brain injury where fluctuating capacity is not characteristic (Halpern & Szmukler, 1997). Furthermore, the notion of advance decision making is typically absent from primary mental health legislation.^4^

SBDs in psychiatry, which invoke precedent autonomy to authorise precommitment to compulsory treatment, were first discussed in the early 80s. Although the idea has been discussed repeatedly and with considerable enthusiasm by both libertarian and conservative voices (Gallagher, 1998, pp. 782–3), this has then been stifled by the numerous rights-based and practical concerns which have emerged within ensuing debates (Dresser, 1982; Miller, 1998). The Netherlands is the only European jurisdiction where this form of precedent autonomy is codified.^5^ Since 2008, the Dutch Psychiatric Hospital (Compulsory admissions) Act – “Wet Bopz” Act – permits a non-capacity based SBD (where implementation does not depend on a judgement of loss of DMC-T) to authorise compulsory treatment if various procedural safeguards are satisfied. There has been no major empirical research into SBDs and, in the Netherlands, their usage has been extremely limited and generally impracticable, due to the extent and complexity of the surrounding legislative safeguards (Berghmans & van der Zanden, 2012). Given that rights-based concerns remain significant arguments against SBDs, it is interesting to note that their introduction in the Netherlands in 2008 stemmed from service-user demand to give patients more influence over treatment, in response to what were perceived as the limitations of 1994 liberal law reforms. Service-user organisations wished to retain the right to secure compulsory treatment at a lower threshold than the immediate risk-based criteria for commitment dictated (Berghmans & van der Zanden, 2012; Varekamp, 2004).

## 3 Self-binding in bipolar

SBDs are often known as Ulysses Contracts. In Book 12 of Homer's *Odyssey*, Odysseus (Ulysses) tells his crew to bind him to the mast of his ship, so that he can experience the irresistible song of the Sirens without being drawn to self-destruction by abandoning his mission and wasting away on their island, bewitched by the sublime pleasure and enlightenment they offer. Like Odysseus, individuals like P, who experience mania, can anticipate that they will be unable, even through rational persuasion, to prevent themselves from damaging behaviour at some future point and that they will be lacking capacity to make treatment decisions. Despite difficulties, SBDs could have the potential to help patients like P to exert control over this situation (Sarin, 2012).

### 3.1 How would an SBD work?

In order for it to be both practicable and ethically viable, our proposed model of SBD is entirely predicated on the notion of DMC-T and lays out a framework in which SBDs inform and help to determine DMC-T assessment, at the same time as being entirely dependent upon the outcomes of such assessment for the enforceability of the interventions which they request.

SBDs have generally been seen as a sub-type of “instruction” PAD, which request the use of compulsory treatment at an earlier stage of the illness than would conventionally be judged as eligible for compulsory treatment (Gevers, 2002; Halpern & Szmukler, 1997; Sheetz, 2006; Varekamp, 2004). This has led to a widespread assumption that such directives are ‘competence insensitive’ and could enable the use of compulsory treatment while an individual still has ‘competence’ or capacity. Gallagher, for example, states that ‘the sine qua non of a Ulysses clause is its attempt to provide for the irrevocability of an advance directive even during a time when the declarant is technically competent’ (Gallagher, 1998). This assumption has, understandably, fuelled many of the strongest concerns about such measures (Bielby, 2014; Davis, 2008; Dresser, 1982; Gallagher, 1998; Radden, 1994; Walker, 2012).

However, for an SBD is to be ethically viable, it is critical that it does not violate the principle that those with capacity to make autonomous decisions must be allowed to do so freely and that anyone with DMC-T must be permitted to revoke or revise such an agreement (Atkinson, Garner, & Gilmour, 2004; Berghmans & van der Zanden, 2012; Gremmen et al., 2008; Halpern & Szmukler, 1997; Sheetz, 2006; Widdershoven & Berghmans, 2007). Accordingly, our suggested model of SBD does not simply request particular treatments when particular behaviours are witnessed, but takes these behaviours to be key indicators that DMC-T may have been lost and requires assessment. Unlike other advance directives, which are only invoked after a patient has already been judged to be lacking in DMC-T, SBDs would thereby have an informing role in the assessment of capacity, as well as directing any compulsory treatment once capacity is judged to be lost.

In order to work effectively, an SBD would involve the following type of process:

1) Assessment that the individual is eligible, insofar as: a) they currently have capacity to evaluate their future healthcare needs in the light of relevant information and experience; b) they are making a free and informed choice, without any pressure or coercion; c) they are creating a directive for treatment recommended by an appropriate clinician, based on clear past experience of prior episodes, consequences and treatment (Gevers, 2002; Gremmen et al., 2008).
2) The individual identifies a typical set of behaviours which can be taken as firm indicators of an incipient and escalating episode of mania with associated harms. They might stipulate further conditions, such as how many of these behaviours must be witnessed before the directive is invoked.
3) The SBD requests that, if they begin to manifest such behaviours, the following process should be followed: a) the behaviours are recognised by the clinical team as likely indicators of an incipient episode and they should be offered the treatment which the clinician and the SBD approves^6^; b) if they refuse treatment, or act in such a way that that treatment is not happening, they should be immediately assessed for the lack of DMC-T; c) assessors must use the behaviours and information given in the SBD to determine whether they consider the individual to be lacking in DMC-T; d) if the individual is found to be lacking in DMC-T on this basis, they should be subject to compulsory detention and treatment *in line with recommendations* which have been clinically approved on the SBD. This line should be followed unless new clinical information has come to light that justifies change in the clinical recommendation. The compulsory treatment would be overseen by Mental Health Act tribunals or courts in the usual way and part of the responsibility of the tribunal or court would be to ensure that the SBD is being respected.
4) The SBD could also include some indication of time needed for compulsory treatment, preferred treatment options were first-line treatments to fail, behavioural indicators which should lead to termination of compulsory treatment and indicators for regaining DMC-T. These components should influence the responsible clinician or the tribunal/court without being binding. (Gevers, 2002).
5) As a further point, in order for the individual to have some guarantee that the SBD will be taken into account, we would recommend that there must be some provision for formal recognition of such documents and accountability, if they are either not invoked or not followed.
6) If, and only if, the individual has DMC-T, they are free to revoke or revise their SBD at any time (Berghmans & van der Zanden, 2012; Gevers, 2002).

An SBD would also include certain factors which might otherwise go in instructional directives, such as non-treatment related aspects of care and management of affairs while ill and desired involvement of family members and/or carers. It would thus play a dual role of both guaranteeing treatment, but also influencing care decisions, both aspects of which have been highlighted as important by patient discussions of such interventions (Varekamp, 2004).

Effectively, an SBD would lower the parameters at which to consider introducing coercive interventions and make the threshold for determining loss of DMC-T more tailored to bipolar, but also far more dependent on the individual's own views about how their condition manifests and its probable consequences. Current frameworks of assessment are based too heavily on a decontextualized assessment of behaviours and views regarding treatment at that time. By contrast, SBDs would both require and facilitate a framework of assessment and treatment which accommodates an appraisal of the individual's behaviours, values and beliefs in the broader context of their history and which can allow their own consideration of best interests and potential harms to have a determinative role in their treatment. “Lack of attention to the patient's underlying values and beliefs” has been identified as a shortcoming of advance directives in general, with supplementation with a type of “values-history” proposed as a solution (Berghmans & van der Zanden, 2012).

In broad terms, this is similar to the Dutch SBD model. However, rather than being integrated into an existing capacity or mental health assessment, the Dutch SBDs are subject to distinct and complex legislation, requiring extensive legal and clinical involvement for both construction and enforcement. As mentioned above, this has made them unfeasible. Despite reforms, take-up is extremely small and may even be decreasing (Berghmans & van der Zanden, 2012). Moreover, although proponents recognise the necessity of lack of DMC-T for the SBD (Berghmans & van der Zanden, 2012; Gremmen et al., 2008; Widdershoven & Berghmans, 2007), and the law itself stipulates that the patient must have DMC-T when writing the SBD (Berghmans & van der Zanden, 2012), the Dutch model appears to base the decision to use involuntary treatment simply on the subsequent presence of prodromal behaviours, rather than a judgement of loss of DMC-T. In an example given by Berghmans and van der Zanden an SBD authorised compulsory detention and treatment for 6 weeks, for a woman with bipolar if at least two out of eight prodromal indicators occur. These included unusual expenditures, inviting people for dinner and buying presents, declining self-care (e.g. smell of urine) and “basic rejecting attitude”. However, without subsequent capacity assessment, a significant overlap with normal behavioural variation leaves the Dutch SBD open, not only to the problems of over-juridification or complications, but to ambiguity and the concomitant rights-based concerns about whether the person subject to involuntary treatment is, in fact, really lacking in the ability to decide treatment for themselves.

### 3.2 Potential advantages of an SBD?

For the individual and those around them, the clearest advantage and primary motivation for an SBD would be damage limitation (Atkinson et al., 2004; Berghmans & van der Zanden, 2012; Dresser, 1982; Gallagher, 1998; Gremmen et al., 2008; Sheetz, 2006; Varekamp, 2004; Widdershoven & Berghmans, 2007). P, for example, wants to know that he can protect himself from the personal damage and loss of property which result from his episodes of mania. This would bring with it, not only significant material advantages, but a sense of control over illness and life. More expeditious treatment may result in shorter, less severe or more easily treatable episodes. It is even possible that less severe attacks might, ultimately, lead to greater overall stability and improved health outcomes. Given that the SBD also identifies treatments and management deemed most desirable and acceptable to the patient, while also building trust, it might be the case that SBDs will lessen the level of perceived or actual coercion within treatment (Gremmen et al., 2008; Varekamp, 2004). Equally, the patient, may be able, especially in the earlier stages of an episode, to comprehend the need to accept treatment based on the existence of the SBD, even if the SBD is never formally invoked, so that use of formal compulsion is avoided.

Drafting an SBD would create an opportunity for detailed reflection and engagement between the patient and their clinical team. Both may feel that it enables the patient to take greater responsibility for their care, while also increasing levels of trust and awareness between them, with increased communication and trust frequently identified as a major benefit of both SBDs and PADs in general (Halpern & Szmukler, 1997; Sheetz, 2006; Swanson et al., 2003; Varekamp, 2004; Weller, 2013). The wider benefits which an increased sense of engagement, responsibility and regained control could bring to the patient should not be underestimated.

A condition like bipolar, where capacity can fluctuate, where a person can truly gain experience of the difference between their preferences when manic and when not, and where they can be in a position to evaluate that difference, seems eminently suited to precedent autonomy. SBDs could be one way towards making treatment for severe psychiatric illness more harmonious with the broad aspirations of the CRPD.

## 4 Potential difficulties of SBD and how to address them?

Difficulties surrounding SBDs can be divided into two sets of concerns: ethical ‘rights-based’ and clinical. In this section we set out some of the most significant of these difficulties and offer suggestions for moving towards some workable solutions. As we stated initially, we are far from suggesting that SBDs represent either a perfect or universally appropriate solution for those affected by mania. Neither human rights nor clinical realities are straightforward, and this is especially the case in a scenario such as P's, enmeshed as it is in a set of often conflicting priorities: his own desire to protect himself; his resistance to treatment when manic and the subjective distress brought by enforced treatment; the moral obligation to safeguard his rights to liberty and autonomy; the practical limitations of the clinical situation. Nevertheless, the absence of a perfect or infallible solution in this context should not deter us from attempting to find the answer which is, on balance, the most ethically and practically coherent for the individual themselves. Indeed, we think that cases such as P's should stimulate the development of human rights and clinical thinking.

As well as ethical and clinical concerns, there are, of course, the practical questions of statutory drafting and clinical implementation. Our aim here, however, is a more preliminary and general consideration of how an acceptable SBD might be constructed, without making any precise legislative suggestions.

### 4.1 Ethical “rights-based” questions

#### 4.1.1 Writing the SBD — free and informed consent

The main worries surrounding informed consent are how to ensure that the individual has capacity for informed and autonomous decision-making when drawing up an SBD and to ensure that the individual has made a truly free choice to write an SBD, without feeling pressurised or coerced in any way.

Informed consent depends upon DMC-T and we, like others, regard this as a prerequisite for drafting an SBD. The nature of fluctuating capacity in a condition such as mania means that, in theory, establishing a period of DMC-T to construct the SBD during remission should not be problematic. SBDs rely on the premise that the patient fully recovers DMC-T between episodes, which enables proper reflection and decision-making. Bipolar is a condition which has shown to be associated with full recovery of DMC-T (Owen et al., 2011). As the case of P clearly shows, there seems no reason to see insufficient capacity as a probable stumbling block. Equally, SBDs must also fulfil the criteria for informed consent by ensuring that patients with capacity understand they are free to revoke or revise.

It is also possible that worries about the lack of capacity or general ability to engage in the necessary considerations stem from views that psychiatric patients, even when well, are more vulnerable than others or that their judgement is in some way always compromised (Sarin, 2012; Sheetz, 2006). This can give rise to a type of ethical protectionism, which ends up limiting patients' choices, through false assumptions about their capabilities for decision-making. So, even Dutch proponents of SBDs reflect on the worry: “what are acceptable ways of discussing a SBD with a mentally ill patient, given their vulnerability? (2012)”. Once again, if we consider P, we see a deeply intelligent man ready to face the harsh realities of his condition and suggest what will be, for him, a tough but coherent solution — a very different picture of an individual from the psychological and intellectual fragility often ascribed to those with mental disorders.

Informed consent for treatment requires that a patient has sufficient information about the process to which they are consenting. Here, consent to an SBD may be seen as more ‘informed’ than many other treatment decisions because it is based on the patient's own prior experience of both the condition and the treatment (Cuca, 1993; Gevers, 2002). Unlike advanced refusals and instructional directives, which can be constructed independently of the clinician–patient relationship, SBDs depend upon involvement with a clinician who has a full understanding of the individual case history and is able to make appropriate recommendations.

Although we present this potential expansion of the clinical relationship as a positive (Gevers, 2002), one of the most pervasive and strongest concerns surrounding SBDs is the question of how to ensure that consent is given voluntarily, without pressure or coercion from either clinicians or carers (Atkinson et al., 2004; Berghmans & van der Zanden, 2012; Cuca, 1993; Dresser, 1982; Gevers, 2002; Varekamp, 2004). Clearly, the introduction of SBDs would need a provision to ensure that no type of undue influence is exerted, as has been established within the Dutch SBD laws. However, the solution is not simply to shift power away from the clinical relationship.

It is often argued that even collaboration between patient and team takes place within the power-structure of a “coercion context”:

The very possibility that coercive measures can be used will be part of the situational context in cases in which staff and patients differ in their opinions about what is the best course of treatment to undertake. Hence, there is a subtle interrelationship between coercion and compliance in all realms of psychiatric care (Sjöström, 2006).

Nevertheless, the “coercion context” remains implicit in any discussion of substitute decision making. Arguably, at present, the predominant power structure lies not so much within the clinician–patient relationship, but within risk-based mental health laws coupled with inaccurate public perceptions of the dangers of those with mental illness. The disadvantages of such laws, both for patient rights and for the clinician–patient relationship, has been well examined elsewhere (Dawson & Szmukler, 2006). It may well be that shifting more power towards the clinician–patient relationship, removed from social perceptions of risk, could in fact serve to protect patients from more malign power structures in relation to writing SBDs. At present, patients are subject to ever present risk of repeated compulsory treatment in which SBDs cannot play a role. For these reasons we, like Widdershoven and Berghmans (2001), consider that the writing of an SBD needs to be a collaborative process between clinician and patient, which make specific and mutually acceptable recommendations based on past experience of treatment.

An alternative possibility of creating a formal role for a carer or nominated non-clinical advocate in drafting an SBD and sanctioning enforcement seems problematic. Undue pressure from relatives and partners themselves is often cited as a concern surrounding SBDs (Varekamp, 2004) and, unlike clinicians, carers, or legal advocates, can lack clinical understanding and are removed from systems of treatment delivery and clinical governance. Finally, there is also the danger that in the course of a manic episode, where the proxy themselves may be the object of patient's manic thinking and feeling, such a role could put unfair, or extreme, pressure on relationships serving a non-coercive function (Atkinson et al., 2004).

Even though patients may recognise that SBDs bring a risk of undue influence within the clinician–patient relationship, they can still view the lack of an SBD provision as restrictive and damaging, as was the case in the Netherlands and is reflected in the desires expressed by P. Creating safeguards would be a difficult balance between ensuring both protection, but also that such protection does not stifle the practicability of creating SBDs. It would seem unjustified to reject *tout court* a potentially valuable intervention due to fears of abuse of power within the clinician–patient or other relationships (Sheetz, 2006). Such risks can never be entirely avoided. However, this should not stand in the way of seeing the increased level of choice and freedom an SBD might bring to an individual's life, by giving them the freedom to plan for the future, knowing that they have taken steps to insure themselves against the repeated devastation their episodes of mania can bring.

#### 4.1.2 Enforcing the SBD

Once an SBD is drafted, there are a number of major concerns that can be raised surrounding enforcement. First, even though we consider loss of DMC-T as a necessary condition for enforcement of an SBD, concerns may still remain about the objectivity of DMC-T assessment. Next, there are concerns about the justifications for use of compulsory treatment: can the harms of mania ever be sufficient to justify compulsory treatment and are there sufficient moral grounds for the person with bipolar, when well, to exercise precedent autonomy over themselves, when manic (Sarin, 2012; Van Willigenburg & Delaere, 2005)?

Before addressing these questions, let us first hear from P himself, in his non-manic state, reflecting on his experience when manic: “what you've been doing was chaotic and uncontrollable”; “when you're high, you're not in control of your faculties fully…you don't make the right decisions”; “your decision-making becomes erratic, whereas when you're normal you wouldn't put yourself in them type of jeopardies”; “you cross the bridge in your mind. You cross the bridge where you're no longer rational”.

On the objectivity of DMC-T assessment, first and foremost comes our suggestion that DMC-T should be informed by the individual's SBD, with its detailed and specific indicators of loss of DMC-T, based on approved clinician–patient analysis of case history (Gevers, 2002).^7^ In more general terms, research into DMC-T in mania is in its infancy and the concept itself is complex, due to its crossover between psychiatry, law and ethics. Even so, when judgements of DMC-T are structured in settings where mania is common, interrater reliability is very good (Cairns et al., 2005b). Further development of tools for DMC-T assessment specifically tailored to mania and its preceding and subsequent states (“hypomania”) would help to refine understanding of DMC-T and be useful for creating effective SBDs.

Despite continuing debate, public policy, as reflected in mental health laws, accepts the use of compulsory treatment in all liberal democratic societies of which we are aware. Is this then justified in the case of mania? The harms of mania are real, well documented, and have been directly experienced by those who are considering an SBD. P, for example, judges the self-inflicted damage to himself when manic as a direct consequence of the mental disorder he himself is unable to control. Moreover, a precondition for drafting an SBD must be a judgement agreed by both patient and the clinician that harms consequent to the mania justify compulsory treatment. Thus, the appraisal of harms is person-centred, rather than external, and compulsory treatment is effectively self-sanctioned. In such a context, objections to compulsory treatment for mania risk denying a sufferer, who has both DMC-T and personal experience of mania, the authority to know what represents their own interests with regard to compulsory treatment. Such objections, based as they are on the prioritisation and protection of individual autonomy within mental healthcare now appear self-contradictory and ethically untenable.

Are there moral grounds for appealing to precedent autonomy within bipolarity? Does the autonomy of P when well trump the will and preferences of P when manic (Dresser, 1982; Sarin, 2012; Sheetz, 2006; Van Willigenburg & Delaere, 2005)? If we respect the person by respecting the person's will and preferences then how can we respect both the person at time 1 (when well and wanting to write a SBD) and at time 2 (when manic and refusing treatment)? These questions are philosophically complex but we may start to see a way through them by looking again at P's predicament.

Precedent autonomy, as reflected in an advance directive, relies upon a combination of continuity and discontinuity of personhood, together with a loss of DMC-T at the time when the directive is invoked. As reflected in his metaphor of the bridge between irrationality and ‘normality’, P views his manic self as fundamentally different from his well self in terms of his values and beliefs such as responsibility towards his family, regard for his own safety and property and religious views. This change is not a complete transformation of personhood. Even when manic, P continues to recognise himself as P and have a memory of his life, including previous manic episodes. Yet he lacks his usual inhibitions, gives away all his possessions and enters into situations which he would ordinarily recognise as enormously risky. Moreover, with fluctuating capacity change is not irreversible and directives present views both before and after loss of capacity, meaning that concerns over permanent change of personhood, which feature in debates on advance directive for irreversible illness contexts have minimal bearing here (Buchanan & Brock, 1989; Davis, 2002).

P himself talks about his absence of DMC-T when manic. He sees his manic self as uncontrollable and not subject to any internal conflict or inhibitions which might prevent him from acting on the basis of his ‘well’ values and beliefs. Discussions of SBDs often draw on the misleading parallels taken from discussions of precommitment as a means to control ‘weakness of will’ or *akrasia*, such as Christmas savings accounts which impose penalties on an unrestrained spender for early withdrawal or Elster's well-known example of the lecherous academic, who takes his wife to a faculty party, so that he will be discouraged from too much drink or flirtation (Elster, 2000, p. 11). Not only do such examples misrepresent the impact of mania, they depend upon the presence of synchronic conflicting desires and the possibility of rational contemplation and dissuasion.^8^ Yet the notion of mania invoked within an SBD is more akin to the overwhelming bewitchment of the Sirens, which leaves its listener utterly powerless to resist being lured towards destruction or to recollect their ordinary values and priorities in a way which could function as some type of rational disincentive.

*Akrasia* also implies some intrinsic weakness of character. By contrast, an SBD relies on the notion that manic urges result from a disorder extrinsic to the values, behavioural patterns and beliefs constitutive of the patient's true self. SBDs are therefore predicated on a *diachronic* notion of personhood, with which one's true or ‘normal’ self depends on some degree of consistency, or recognisability, over time, and which can be interrupted by the major changes in values and beliefs accompanying mania. Such a model is closer to Davis' suggestions for SBDs, in which the fact that non-manic wishes are diachronically dominant justifies the applicability of precedent autonomy to mania (2008)^9^. It should not be taken, however, to imply a strong notion of teleology or narrative.^10^

It is also important to differentiate between what we might term “subjective” and “objective” personhood. By subjective personhood we refer to the self as constituted by psychological continuity of values and beliefs, usually referred to as ‘personal identity’ within philosophy. It is this subjective personhood to which we refer as both dependent upon diachronic consistency or recognisability and which can be compromised or interrupted in certain respects at certain times. By contrast, with objective personhood we mean a third-person view of “person” as legal individual, entitled to human rights. While self-determination or personal autonomy is essential to subjective personhood, objective personhood does not depend upon self-determination, temporary or fluctuating loss of capacity, whether through mania, other forms of mental disorder, drunkenness or physical illness. Objective personhood remains uncompromised by these perennial human phenomena, ensuring that, even when subjective personhood is compromised and DMC-T lost, the individual consequently subjected to the enforcement of an SBD is entitled to protection of their human rights and respect for their first-person experiences as being essentially human.

SBDs rely on these dual notions. Assessment using an SBD would be able to build a picture of whether an individual's values and beliefs are indicative of illness and loss of DMC-T, using evidence drawn from their history and from their very own capacitous appraisal of their values and beliefs and how the current behaviours represent, on balance, a major and self-inconsistent shift which puts them at risk. Misleading suggestions of capacity, which might arise through the synchronic consistency of values, beliefs and behaviour, which can occur, especially during the early stages of mania, may be doubted through their diachronic inconsistency or non-recognisability (Banner & Szmukler, 2013).

This subjective/objective personhood distinction also takes us some way towards addressing challenges stemming from the CRPD's notion of universal legal capacity and the delinking of legal and mental capacity (Richardson, 2012). SBDs seek to protect an individual's subjective sense of personhood and maximise self-determination, through providing them with a means to control their own actions when they lack DMC-T, without compromising legal capacity and entitlement to human rights. Objective legal personhood should accommodate a person's right to view their own subjective personhood as compromised or interrupted at points and their requests for others' assistance in exerting self-directed control over themselves at these times.

#### 4.1.3 Clinical questions

Surveys of mental health clinicians' views on advanced refusals, instructional directives and health proxies reveal a mixed picture; confusion about the law is common, as are concerns about resources or detrimental use of advance refusals (Atkinson et al., 2004; Elbogen et al., 2006; Kim et al., 2008; Van Dorn et al., 2006)^11^. One recent trial of a form of instructional directive (crisis cards for patient with psychosis involving an independent facilitator) reported that clinical resistance during the trial was significant (Thornicroft et al., 2013), whereas a small study of Dutch psychiatrists reported only 2 out of 17 were absolutely opposed to SBDs and many had positive views — especially in disorders with a cyclical pattern (Varekamp, 2004). One qualitative study conducted when the Dutch SBD laws were introduced reported this interesting reaction from a psychiatrist, which highlights how an SBD can, in itself, bring positive changes to the structure and process of the clinical relationship:

“initially I thought [an SBD arrangement] primarily had a juridical, contractual tone… [I was] surprised that something quite different was set in motion… It [the something quite different that was set in motion] may not be primarily what a [SBD arrangement] is meant for but it is a definition of the treatment relationship in terms of mutual obligations (Gremmen, 2008).”

Very little is known about clinical views on SBDs in Bipolar Affective Disorder. This needs further investigation, given that the effectiveness of SBDs would, we argue, depend upon their integration into the clinician–patient relationship to work. We now briefly consider and respond to some concerns clinicians might have about SBDs in Bipolar Affective Disorder.

The lack of time and resources is a significant obstacle. Drafting an SBD would be fairly labour intensive and, at a time when mental health services often feel stretched beyond capacity, making space for incorporating yet another set of processes and regulations might be considered impracticable in some service settings. The CRIMSON study, for example, found that many teams did not set aside specific dedicated time for working on the PADS or that, if they did, not all relevant team members were present (Thornicroft et al., 2013). The fact that Dutch SBDs are considered to be ‘a time-consuming and complex endeavour’ has been identified as a probable contributory factor to their poor take-up (Berghmans & van der Zanden, 2012). Moreover, an effective SBD would require clinical analysis of the illness trajectory within the broader context of the patient's life. This would need to take place during remission, when minimal time is often allocated for clinical involvement, especially from doctors, and it would also require a continuity of care often unavailable.

SBDs change the conventional nature of the clinical relationship, allowing not only more space for communication, but creating a more shared process of decision-making, which allocates more control and responsibility to a patient in remission. These changes frequently cited amongst the most significant potential benefits of PADs would also allow the clinical team greater opportunity to distinguish a patients' underlying personality from their behaviours when ill. Nevertheless, these changes might also provoke some degree of anxiety and uncertainty. In practical terms, clinicians may worry about the rectitude of endorsing this policy, if their services cannot provide the time and resources necessary for the level of continuity of care which the drafting and implementation of SBDs would require.

There may be further concerns about more subtle psychological factors. Clinicians may well feel that retrospective analysis of manic episodes and direct communication about compulsory treatment may be not only time-consuming, but also an uncomfortable process for both patient and clinician. Psychological responses such as denial, projection and avoidance are common coping mechanisms used by patients to deal with experiences which they may find emotionally overwhelming or very threatening to self-esteem. Although clinicians may, as we have already discussed, overestimate the psychological vulnerability of their patients, the type of analysis required for an SBD could be unsettling and it is important that matters of practice are handled with sensitivity. It is important to recognise that SBDs are not an appropriate universal policy to be urged on all those who have experienced manic episodes and that identifying the appropriate time to consider drafting an SBD is critical. We suggest that the impetus for an SBD should come predominantly from the patient, with a clinician able to listen to and supportively guide the patient as the impetus forms. There is also the risk that comorbidity between bipolar disorder and addictions or personality disorders may lead to dysfunctional motivations for drafting SBDs. However, if SBDs must be subject to clinical recommendation and approval, as we suggest, it will be part of the duty of care of the expert clinician to recognise such potential problems.

While all these concerns are legitimate, they may become a self-fulfilling prophecy, if they are left unchallenged, and if mental health stakeholders do not make a strong case for the necessity of continuity of care and allocation of adequate time and resources. Moreover, clinicians discomfort in discussing topics such as compulsory treatment or previous manic episodes should not be allowed to take priority over the needs and best interests of their patients. As P makes clear he wants to talk about these topics because he recognises their future impact and cares about his future.^12^

Finally, there are questions of legislation and enforcement. The current legal status of PADs in general seems to be an instance of an “incompletely theorized agreement”, an “agreement maintained by avoiding the question of enforceability”, so that, despite broad agreement as to their usefulness and development in practice and policy, there is no satisfactory legal framework (Weller, 2013, p. 143). Clinicians might be tempted to keep the SBD process informal to avoid the legal burden, relying on good communication between patient and team. However, given that SBDs would involve compulsory treatment within an innovative framework of anticipatory decision-making, it seems that, in order to safeguard both patient rights, there is a need for SBDs to be codified within law. Moreover, the lack of enforcement is a major concern voiced in relation to SBDs (Varekamp, 2004), so that codification could also include provision for safeguarding the rights to appeal against clinical decisions not to follow its recommendations and for protecting clinicians' SBD-based decisions to overrule their patients' wishes when ill (Sheetz, 2006). Even so, given the Dutch experience of over juridification, it seems imperative to keep the SBD as intuitive for clinician and patient as possible and to integrate it, as far as is possible, within existing structures of mental health law and practice.

## 5 Conclusion

Our aim in this article has been to explore the issues surrounding SBDs in bipolar and give preliminary outlines for a model which might be both feasible within a clinical and legal context and ethically coherent. We have argued for a DMC-T based SBD and for the importance of keeping SBDs nested within the clinician–patient relationship.

Mental health care is currently in a process of change. While resources are limited, human rights law and other support for equality in mental health means that notions such as self-determination and decision-making capacity are gaining increasing importance in determining policy and practice. As this happens, it is also becoming clear that use of these concepts has often lacked nuance, provoking concerns over whether too much emphasis on self-determination might in itself be detrimental in a psychiatric context (Lepping & Raveesh, 2014). It is time, then, to refine our understanding of decision-making capacity in specific conditions like bipolar and to bring this together with a broader understanding of self-determination, able to negotiate the challenges of advance decision making.

SBDs for people with bipolar constitute a treatment request which implicates a clinician's duty of care.^13^ Greater understanding of what are perceived to be the major obstacles, benefits and ideal modes of SBD implementation from both a clinical and a patient perspective is therefore required. Furthermore, given that avoidance of undue complexity in codification seems so crucial to the success of introducing and implementing SBDs, there is a need for legal research into how best to achieve this within different jurisdictions. There is a delicate balance to be maintained between safeguarding, practicability and respecting the patients' own voice.

As we proposed at the outset, this can never be an ideal situation. Those who live with conditions such as mania are faced with the dilemma of managing a condition which can cause them, on repeated occasions, to act in a way utterly uncharacteristic and destructive of the things which they value most. In what could be considered the ultimate statement of self-determination, patients like P have acknowledged that they cannot protect themselves from themselves and need external constraint. Like Odysseus, who recognises that he will find the ropes which prevent him succumbing to the Sirens as *argaleos* (‘galling’, ‘painful’, ‘violent’, ‘shocking’ or ‘difficult’), P knows, from repeated personal experience, how painful it will be for him to be treated compulsorily. Yet, he himself feels this would be the best possibility for shielding himself and his family from damage and regaining control over his life. For P, as for so many others throughout the history of this debate, an SBD may well seem the most obvious and compelling way of managing his difficulties.

As Rosenson and Kasten so eloquently argued in 1992, in support of introducing SBDs:

Those individuals struggling with mental illness are heroes committed to their own odyssey. If some can put a protective mechanism in place to make the journey less arduous for themselves, and pave the way for others, rights advocates may come to respect the patient's right to plan and indeed encourage it (1991).

The history of SBDs has been one of support repeatedly stifled by concerns. Even when the Netherlands finally allowed formal codification of SBDs, they have been enmeshed in so many protective measures that they have become almost untenable. Despite the difficulties posed by SBDs, it is time to move forward. Individuals such as P deserve the right to make their own decisions about what is truly in their best interests. Yet, with savage irony, it seems that society's desire to protect P may be the main obstacle standing in the way of allowing him to protect himself.

## Footnotes

1 This authorises compulsory admission and treatment for a period of up to 6 months at first instance with discharge possible at any point by the treating clinician or a tribunal.

2 R v C [2009] UKHL 42.

3 In an example given by Dworkin, a Jehovah's witness' autonomous advance of blood products must be followed when, later, in a deranged mental state, they pleaded for a blood transfusion (p227).

4 For example, in England and Wales it is absent from the Mental Health Act (1983) and therefore has no statutory force although it is present in the Code of Practice for this Act (secondary legislation).

5 As far as we are aware the only state in North America that provides a SBD is Washington State. In 2003 Washington made a provision for advance directives consenting to treatment and waiving the right to refuse treatment based on decision-making capacity (Revised Code of WashingtonTitle 71 — MENTAL ILLNESS 71.32, 2013). Very little is written about implementation. We note that the cognitive understanding of capacity within Washington law (RCW 11.88.010 (1) (e)) is a poor fit for mania and that legal opinion on the Washington SBD appears divided (Anderson, 2003; Sheetz, 2006).

6 These transition points can be subtle and offers of treatment may be accepted without overt requests for them. P says about his transitions “I often know subconsciously when I am unwell, even if I don't admit it — so if I am asking for appointments to see someone that probably means I need to be seen and given some extra support”.

7 Washington and Pennsylvania have allowed an individual to designate indicators of loss of DMC-T on an advance directive (Sheetz, 2006, p. 417).

8 As Aristotle says ‘the akratic acts on account of passion, even though he knows it is wrong’ (*NE* 1145b13). Van Willigenburg and Delaere draw on Elster's inclusion of mental disorder amongst instances of *akrasia*, and class ‘manic psychosis’ amongst instances where ‘passions distort and cloud human reason’ and ‘we sometimes act against our own better reason’ (Van Willigenburg & Delaere, 2005, pp. 397–402).

9 However, Davis sees this as a means to justify the overruling of the patient's competent wishes at this point, rather than suggesting that the inconsistencies between current and diachronic values and beliefs might suggest that competence is in doubt.

10 While an SBD may seem most appealing to those with a more goal-directed or narrative sense of self, it would be perfectly possible for an individual's personhood to reflect diachronic consistency of values and beliefs in the very changeability of their sense of self.

11 Anxiety can be sparked by cases such as *Hargrave v Vermont* 340F.3d 27 (2d Cir. 2003), where Hargrave launched a successful appeal against the Vermont laws utilised in an attempt to overturn her LPA type PAD refusing all psychiatric treatment.

12 It is interesting to note that a Bipolar Service at the Massachusets General Hospital builds anticipatory decision-making into the clinician–patient relationship (ref http://www.medscape.org/viewarticle/571159).

13 Although we have focused on bipolar disorder throughout this article, treatment requests involving self-binding may also come from people without this diagnosis. Future research on SBDs should also focus on the specific clinical and ethical characteristics of other severe mental disorders, such as severe recurrent depression and some forms of schizophrenia and related disorders, which also have a phasic course.
