# Corrigendum to ‘Fluctuating capacity and advance decision-making in Bipolar Affective Disorder — Self-binding directives and self-determination’ [Int J Law Psychiatry. (2015) 92–101]

**DOI:** 10.1016/j.ijlp.2015.06.001

**Published:** 2015

**Authors:** Tania Gergel, Gareth S. Owen

**Affiliations:** a Department of Psychological Medicine, King's College London, Institute of Psychiatry, Psychology and Neuroscience, C/O Department of Classics, King's College London, Strand, London WC2R 2LS, UK; b King's College London, Institute of Psychiatry, Psychology and Neuroscience, Department of Psychological Medicine, UK

The authors regret an omission in the first sentence of 2.3, which should read ‘Advance decision-making can be through of as a codification of what Ronald Dworkin called “precedent autonomy”’.

The authors would like to apologise for any inconvenience caused.

